# Effects of temperature fluctuations on spatial-temporal transmission of hand, foot, and mouth disease

**DOI:** 10.1038/s41598-020-59265-z

**Published:** 2020-02-13

**Authors:** Chengdong Xu, Xiangxue Zhang, Li Wang, Yuke Zhou, Gexin Xiao, Jiaqiang Liao

**Affiliations:** 1 State Key Laboratory of Resources and Environmental Information System, Institute of Geographic Sciences and Natural Resources Research, Chinese Academy of Sciences, Beijing, 100101 China; 2 State Key Laboratory of Earth Surface Processes and Resource Ecology, Beijing Normal University, Beijing, 100875 China; 3 College of Environment and Planning, Henan University, KaiFeng, 475001 China; 4 Key Laboratory of Geospatial Technology for the Middle and Lower Yellow River Regions (Henan University), Ministry of Education, Kai Feng, 475001 China; 5 Key Laboratory of Ecosystem Network Observation and Modeling, Institute of Geographic and Nature Resources Research, Chinese Academy of Sciences, Beijing, 100101 China; 6 China National Center for Food Safety Risk Assessment, Beijing, 100022 China; 7 School of Public Health, Tongji Medical College, Huazhong University of Science and Technology, Wuhan, 430074 China

**Keywords:** Climate sciences, Infectious diseases

## Abstract

Hand, foot, and mouth disease (HFMD), predominantly occurs among infants and children. Previous studies have shown that suitable, stable temperatures favor HFMD virus reproduction; however, temperature fluctuations also affect virus transmission, and there are, so far, no studies concerning the association between such fluctuations and the incidence of HFMD. The objective of this study was to map the spatial-temporal distribution of HFMD incidence and quantify the long-term effects of temperature fluctuations on HFMD incidence in children. HFMD cases in children under five, from January 2009 to December 2013, in Beijing, Tianjin, and Hebei provinces of China, were used in this study. The GeoDetector and Bayesian space-time hierarchy models were employed to explore the spatial-temporal association between temperature fluctuations and HFMD incidence. The results indicate that HFMD incidence had significant spatial stratified heterogeneity (GeoDetector *q*-statistic = 0.83, *p* < 0.05), and that areas with higher risk mainly appeared in metropolises and their adjacent regions. HFMD transmission was negatively associated with temperature fluctuations. A 1 °C increase in the standard deviation of maximum and minimum temperatures was associated with decreases of 8.22% and 11.87% in the risk of HFMD incidence, respectively. The study suggests that large temperature fluctuations affect virus growth or multiplication, thereby inhibiting the activity of the virus and potentially even leading to its extinction, and consequently affecting the spatial-temporal distribution of HFMD. The findings can serve as a reference for the practical control of this disease and offer help in the rational allocation of medical resources.

## Introduction

Hand, foot, and mouth disease (HFMD), a disease that predominantly occurring among infants and children. It is characterized by flu-like symptoms; a rash on hands, feet, and buttocks; mouth ulcers; poor appetite; and vomiting and diarrhea^1,2^. The disease is transmitted from person to person by direct contact with respiratory secretions, or through fecal-oral transmission. HFMD can be seriously life threatening, particularly in patients who rapidly develop neurological and systemic complications, which can be fatal^1,2^.

The primary viruses that cause HFMD are enteroviruses, of which enterovirus 71 (EV71) and Coxsackie virus A16 (CV-A16) are the most commonly reported^2^. EV71—a single-stranded RNA virus that belongs to the same category as poliovirus and was first diagnosed in California in 1967—is commonly related with the most severe symptoms, including central nervous system disorders and even the development of fatal pulmonary edema^1^. Over the past few decades, HFMD outbreaks have been reported worldwide, mainly in Asia-Pacific countries, including China, Singapore, Vietnam, Japan, and Malaysia^3–7^. In 2007 and early 2008, China experienced several large outbreaks of HFMD and promptly created a national enhanced surveillance system in response. Thereafter, on May 2, 2008, HFMD was listed as a category C infectious disease in China, and was made statutorily notifiable. Notably, breakouts of HFMD in many countries have continued to increase due to climate change, viral mutation, lack of comprehensive monitoring systems, and limited medical resources^8^.

**Figure 1:**
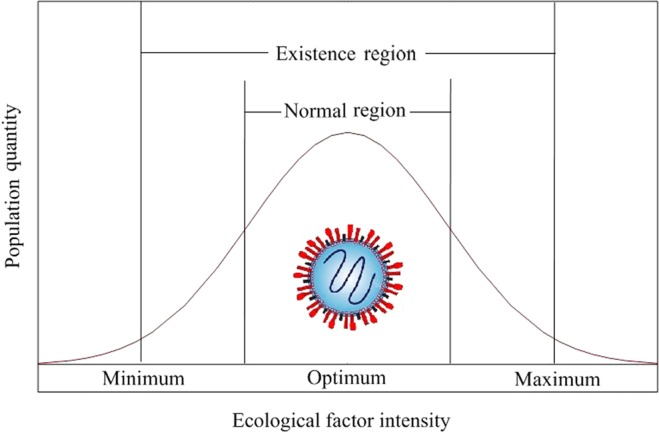
The law of tolerance for HFMD viruses.

The survival of an organism has been affecting by several environmental factors, genetic composition, evolutionary trend, biological composition and so on. And Shelford’s law of tolerance states that an organism can exist and multiply in a suitable, stable environment, but if the variation of an environmental factor exceeds the tolerance of that organism, the species cannot survive, and may even become extinct^9,10^. This applies equally to enteroviruses, such as HFMD viruses (Fig. 1). Epidemiological evidences also show that the spread of HFMD viruses is elevated by meteorological factors^3,5^. For example, Liu *et al*. found that moderate temperatures promote the growth and transmission of the viruses that cause HFMD^11^ and Zhu *et al*. noted that higher temperature promote faster virus reproduction, contributing to increased risk of HFMD incidence^12^. Similarly, previous studies have demonstrated that, when the temperature is above 25 °C, the infectivity and activity of EV71 is restricted^13^, while an *in vitro* experiment found that enterovirus replication was enhanced at 39 °C^14^. Furthermore, in the broader study of virology, there is other evidence showing the temperature-sensitive nature of enteroviruses and other human enteric viruses^15^.

Although it is evident that HFMD virus reproduction and transmission is affected by meteorological conditions, such as temperature and humidity, there remain, to our knowledge, no studies concerned with the association between HFMD and temperature fluctuations that might disrupt the suitable, stable environment for the virus and directly affect its survival, and thus influence HFMD transmission^12–14^. Furthermore, against the background of global climate change, temperature variance has been continually intensifying^16^; thus assessing temperature fluctuations and their relationship to human health could provide novel evidence for policy makers and medical institutions to identify a focus for interventions and the optimal allocation of health resources.

The objective of our study was to 1) map the county-level spatial-temporal distribution of childhood HFMD incidence in the Beijing-Tianjin-Hebei area, China, from 2009 to 2013, and 2) quantify the long-term effects of temperature fluctuations associated with HFMD incidence, while controlling for other factors.

## Results

**Figure 2:**
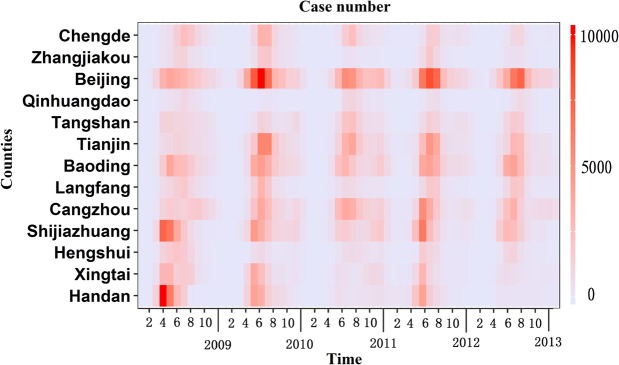
Heatmap of case number in the study area from 2009 to 2013.

Between January 1, 2009, and December 31, 2013, a total of 598,835 cases in five years of HFMD in 208 counties were reported in Beijing-Tianjin-Hebei area. There presented cyclical trend during the study period. The highest number of cases occurred in the late spring and summer (May to July), with a monthly incidence of 41.03 per 10,000 people. The lowest number of cases appeared in winter (December to February), with a monthly incidence of 1.56 per 10,000 people (Fig. 2).

**Figure 3:**
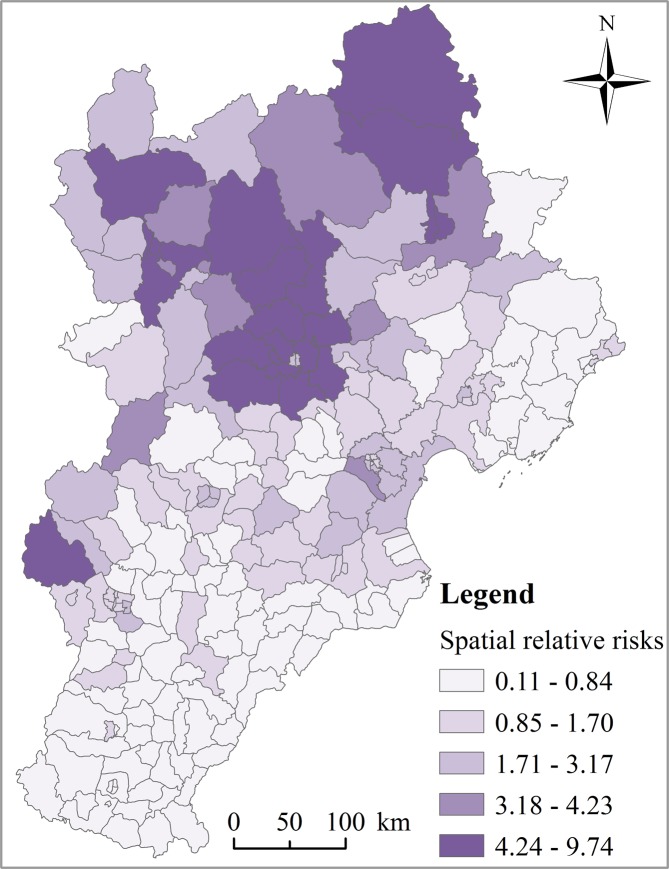
The posterior means of the spatial relative risks (RRs) (exp(*s*_*i*_)) of HFMD for each county in the Beijing-Tianjin-Hebei area.

The relative risk of HFMD varied across the study counties geographically; the GeoDetector *q* statistic value was 0.83 (*p* < 0.05), which indicates that there was significant spatial heterogeneity for HFMD risk. The high-risk areas mainly appear in large cities (e.g., Beijing and Tianjin) and their adjacent counties (Fig. 3).

### Effect of potential driving factors on HFMD epidemics

In the study, the SD of temperature, used as the index of temperature fluctuation, and its effects on HFMD were analyzed using Bayesian space-time hierarchy model (BSTHM). The results showed that a 1 °C increase in the SD of maximum temperature was associated with a decrease of HFMD incidence of 8.22% (95% CI: −14.63 – −1.82), with a corresponding RR of 0.92 (95% CI: 0.86–0.98). The SD of minimum temperature also presented a similar relationship, with a 1 °C increase in SD of the minimum temperature associated with a decrease in HFMD incidence of 11.87% (95% CI: −19.55 – −4.18) and a corresponding RR of 0.89 (95% CI: 0.82–0.96). The effects of SD of average temperature were also assessed, but there was no significant association with the incidence of HFMD (Table 1).

**Table 1:** Quantified posterior means and RRs of BSTHM coefficients.

Variables	Posterior mean (95% CI) (%)	RR (95% CI)
SD of maximum temperature(°C)	−8.22** (−14.63, −1.82)	0.92 (0.86, 0.98)
SD of minimum temperature(°C)	−11.87** (−19.55, −4.18)	0.89 (0.82, 0.96)
SD of average temperature(°C)	6.56 (−4.26, 17.80)	1.07 (0.96, 1.20)
Average temperature (°C)	17.10** (14.84, 19.38)	1.19 (1.16, 1.21)
Relative humidity (%)	3.67** (3.05, 4.30)	1.04 (1.03, 1.04)
Wind speed (m/s)	−17.20** (−26.82, −7.75)	0.86 (0.80, 0.92)
Sun hours (h)	0.06 (−0.07, 0.18)	1.00 (0.99, 1.02)
Precipitation (mm)	−0.15** (−0.22, −0.08)	0.84 (0.77, 1.04)

Note: 95% CI indicates confidence interval with a confidence level of 0.95,

**Statistical significance level: 0.01.

In addition to the temperature variation factor, other risk factors, used as control variables (e.g., monthly average temperature, relative humidity, precipitation, and wind speed), were also analyzed and showed significant relationships with HFMD (Table 1).

A positive association was found between average temperature and HFMD incidence, which was different from the quantitative relationships between the SD of temperature and the disease. A rise in temperature of 1 °C was associated with an increase of 17.10% (97.5% CI: 14.84–19.38) in the incidence of HFMD; the corresponding RR was 1.19 (95% CI: 1.16–1.21). There was also a positive association between HFMD incidence and relative humidity—a 1% increase in relative humidity was associated with a 3.67% increase (95% CI: 3.05–4.30) in the incidence of HFMD, with a corresponding RR of 1.037 (95% CI: 1.03–1.04). Precipitation and wind speed showed negative correlations with HFMD—a 1 mm reduction in precipitation and a 1 m/s decrease in wind speed were associated with 0.15% (95% CI: −0.22 – −0.08) and 17.20% (95% CI: −26.82 – −7.75) decreases in the incidence of HFMD, respectively, with corresponding RRs of 0.86 (95% CI: 0.80–0.92) and 0.84 (95% CI: 0.77–0.93) (Table 1).

## Discussion

HFMD has become an increasingly significant health problem among children in recent years. In this study, the spatial-temporal distribution and effects of temperature fluctuations on HFMD incidence were quantified for the Beijing-Tianjin-Hebei area, China. The study found that there was a significant spatial heterogeneity for HFMD risk, and that disease transmission had a negative association with temperature fluctuations.

Studies have indicated that there is a suitable, stable temperature for transmission of viruses related to HFMD^3,6,12^. Stantonet al. demonstrated that, compared with replication at 37 °C, enterovirus replication was inhibited by nearly 90% at 39 °C, greatly reducing the incidence of HFMD^14^. Onozuka *et al*., in Japan, found that the effect of temperature on HFMD cases peaked at 29 °C^6^. In the current study, it was indicated that temperature change was closely related to disease risk variant, which is similar to the previous studies. For examples, Zhang *et al*. found that, in Henan province of China, the disease of HFMD risk rise 4.09% with a 1 °C increase in temperature^17^. Moreover, Dung *et al*. demonstrated that in Vietnam the HFMD risk increase 7% with a 1 °C rise in temperature^18^. These studies suggest that if the temperature diverges too much from the suitable temperature, it can affect the transmission of HFMD.

To our knowledge, some studies have indicated that temperature fluctuation was a serious health risk factor for the infectious diseases. For examples, Joshi *et al*. showed that, in Korea, the aseptic meningitis has a closely association with temperature fluctuation^19^. Similarly, Beck *et al*. demonstrated that temperature fluctuation was significantly connected with malaria within the malaria transmission zone in sub-Saharan Africa^20^. Meanwhile, Abbas *et al*. found that, in Karachi, temperature fluctuation presented significantly association with dengue fever^21^. Additionally, Joshi *et al*. presented that, in Korea, temperature fluctuation closely related to hemorrhagic fever^22^.

The study found that the HFMD risks also were strongly related to temperature fluctuations, which presented different quantitative relationships compared with that between the disease and average temperature. It was indicated that a 1 °C increase in the SD of maximum and minimum temperatures was associated with decreased risk of HFMD incidence of 8.22% and 11.87%, respectively. The potential mechanism may be because HFMD virus growth and reproduction have adapted to the most suitable temperature, if a large temperature fluctuation occurs, this would exceed the tolerance of virus growth or multiplication and inhibit the activity of the virus or even cause its extinction^9,10^, ultimately affecting the incidence of HFMD.

Moderate and stable temperatures in the environment may be good for the survival of HFMD viruses, accelerating their transmission^11,12^, while large fluctuations of temperature may weaken the reproductive capacity of infectious pathogens and vectors, thus altering the survival of viruses in the physical environment and decreasing disease prevalence accordingly. This is reasonable according to experimental findings that indicate that, when the temperature is higher than 25 °C, the activity and infectivity of EV71 is restricted^13^. A study by Shelford also demonstrated that if the quantity (or quality) of a factor is insufficient or excessive for an organism to exist and multiply, the species cannot survive, and may even become extinct^9,10^. A further study demonstrated that serological antibodies in the human body may adjust according to temperature changes^23^, and thus environmental temperature fluctuations may be one of the major underlying factors influencing the incidence of this disease. To assess the influence of temperature fluctuations, other potential meteorological factors were used as control variables. A positive association was found between relative humidity and HFMD incidence, which is consistent with the results from previous studies. For example, one study indicated that every 1% increase in relative humidity was association with a 4.7% rise in HFMD, and found a threshold of humidity at 80%^6^; another study observed that increased relative humidity was related to a 13% increase in the risk of HFMD^23^.

The current study found that wind speed and precipitation were negatively correlated with HFMD incidence, which is also consistent with other studies. For example, a previous study in Hong Kong found a negative association between HFMD incidence and wind speed^24^, while a study in Singapore reported a negative relationship between precipitation and HFMD^25^, as heavy downpours can disrupt the survival environment of viruses^26^. Another study found that heavy rainfall is negatively related to physical activity^27^, thus significant precipitation could interrupt transmission by reducing social contact^24^.

The results from the BSTHM showed that the spatial distribution of HFMD risk was non-homogeneous. Notably, areas with the highest incidence were mainly concentrated in large cities and their adjacent areas. The areas with developed economies, high population density, and mixed socio-human environment (e.g. Beijing and Tianjin) were the main epidemic regions, which is consistent with previous studies. For instance, a previous study found that the HFMD incidence in economically developed areas, including Beijing, Tianjin, Shanghai, and Zhejiang, was higher than in less developed areas^17,28–30^. A further study found that the incidence of HFMD was higher in the provincial capital city of Chengdu than in other counties in Sichuan province, China^31^. A potential mechanism may be that, due to rapid economic development and urbanization in recent years, there is both higher population density and an increased floating population in large cities and their adjacent counties, thus inevitably promoting more frequent communication and contact with others, which is conducive to the transmission of HFMD.

There are some limitations to this study that should be mentioned. Spatial data at the county level was used, which could introduce an ecological fallacy. Spatial heterogeneity also exists within a county area; for example, populations in urban and rural areas may have different living conditions and healthcare, which would reflect on temperature variations differently. In future studies, data at a finer spatial scale (e.g., villages and towns) will be collected to analyze the relationship between HFMD and environmental factors. Furthermore, the data used in this study was from 2009 to 2013 in Beijing, Tianjin and Hebei province of China, although the findings and conclusions were applicable to the time period and region, in the future, the data covering more regions in recent years will be collected and analyzed to strengthen the study.

## Conclusions

Temperature fluctuations play an important role in shaping HFMD spatiotemporal patterns. HFMD transmission is negatively associated with temperature variations, implying that the risk of HFMD decreases in temperature-unstable environments. These findings can serve as reference and basis for the surveillance and control of the disease in practice, and may be helpful in the rational allocation of medical resources.

## Methods

### Study area

**Figure 4:**
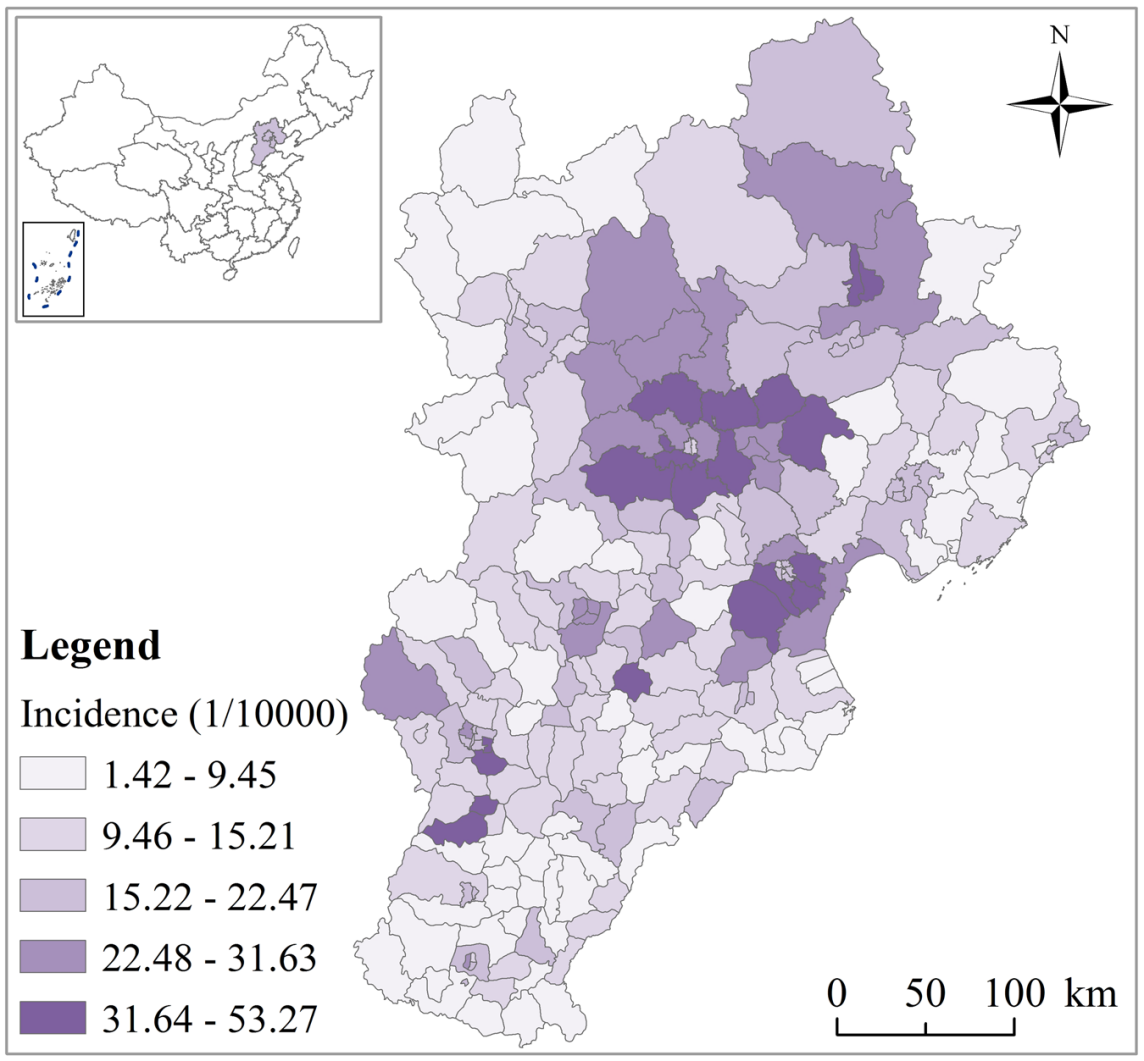
Geographic location of the Beijing-Tianjin-Hebei area in China, with average monthly incidence of HFMD in children from 2009 to 2013.

The study region is located in the north of China; includes Beijing, Tianjin, and Hebei provinces; and is a typical semi-humid continental monsoon climate in the North Temperate Zone. It is hot and rainy in summer, and cold and dry in winter. The geographical location of the study area is shown in Fig. 4.

Beijing is the capital of China, a megalopolis, with an area of 16,400 km^2^ and a population of 20.7 million. Tianjin is a municipality directly governed by the central government, with an area of 11,900 km^2^ and a population of 14.1 million. Hebei province is located around Beijing and Tianjin, with a total area of 188,800 km^2^ and a population of 72.9 million.

### Data

**Figure 5:**
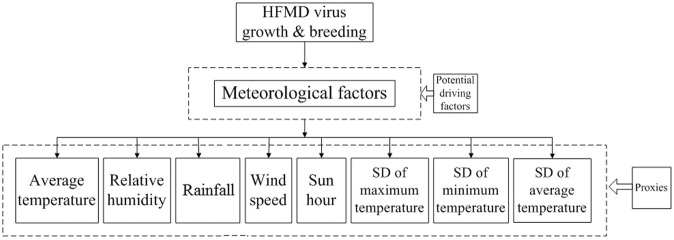
Potential driving factors and Proxies of HFMD.

**Figure 6:**
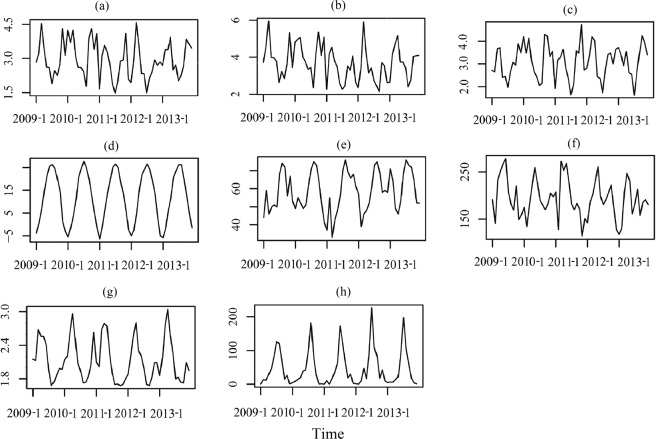
Monthly average value of meteorological factors from 2009 to 2013. (**a**) SD of mean temperature (°C). (**b**) SD of maximum temperature (°C). (**c**) SD of minimum temperature (°C). (**d**) Mean temperature (°C). (**e**) Relative humidity (%). (**f**) Sun hour (**h**). (**g**) Wind speed (m/s). (**h**) Precipitation (mm).

Data on HFMD cases in children under five, from January 2009 to December 2013, was obtained from the Chinese Center for Disease Control and Prevention (http://www.phsciencedata.cn). Monthly meteorological data was obtained for the same period from the China Meteorological Data Sharing Service System (http://data.cma.gov.cn/). Temperature fluctuations were measured by the monthly standard deviation (SD) of average temperature, calculated using the daily average, maximum, and minimum values. In addition to these factors indicating temperature fluctuations, referencing the previous studies, other meteorological factors, including monthly average temperature, relative humidity, precipitation, hours of sunshine and wind speed, were used as control variables in the model (Figs. 5 and 6).

### GeoDetector

GeoDetector is a novel spatial variation analysis method, which can be used to explore the stratified heterogeneity of a responding variable, where phenomena within strata are more similar than between strata^32–34^. In this study, GeoDetector was used to quantify the heterogeneity of the temporal and spatial variations of HFMD risk.

The *q* value can be expressed as:

1 $$q=1-\frac{1}{N{\sigma }^{2}}{\sum }_{h=1}^{L}{N}_{h}{\sigma }_{h}^{2}$$

where *q* denotes the degree of stratified heterogeneity for the dependent variable. Its value ranges from 0 to 1—the larger the *q* value, the more significant the spatial heterogeneity from the target variable. *N* is the number of counties and *σ*^2^ expresses the variance across all the statistical units in the study area. The study area is stratified into *L* strata, presented by *h* = 1, 2,…, *L*, implemented through a discretization process. σ_*h*_^2^ is the variance within stratum *h* in the study area.

### Bayesian space-time hierarchy model

The BSTHM has been widely used in disease mapping with sparse data. This model can overcome, to a certain extent, the shortcomings of a small sample and the autocorrelation of spatial-temporal data. And it also can make full use of the overall information, sample information, and prior information to estimate the posterior distribution of spatial-temporal parameters.

In this study, we used the BSTHM to analyze the county-level spatial-temporal distribution of HFMD incidence from 2009 to 2013 and quantify its relationships with temperature variation, after controlling for other meteorological factors. A space-time hierarchy model with Poisson distribution was used to model the monthly cases of HFMD. Letting *y*_*it*_ and *n*_*i*_ represent the number of disease cases and the risk population, respectively, with county *i* = (1,…,208) and month *t* = (1,…,60), the disease cases can be modeled as:

2 $$\begin{array}{rcl}{y}_{it} & \sim  & Poisson({n}_{it}{u}_{it}),\\ \log ({u}_{it}) & = & \alpha +{s}_{i}+({b}_{0}{t}^{\ast }+{v}_{t})+{b}_{1i}{t}^{\ast }+{\beta }_{v}{x}_{vit}+{\sum }_{n=1}^{N}{\beta }_{n}{x}_{nit}+{\varepsilon }_{it}\end{array}$$

where *u*_*it*_ indicates the potential risk of HFMD in region *i* and month *t*. Among them, *α* is the overall log disease risk during a selected period in the study area. The spatial index, *s*_*i*_, throughout the total study period, denotes the residual spatial distribution of disease risks across the study area; this was affected by some temporal relative stable factors in the study period, such as local geographic environment, economic conditions, and medical resources. Time span relative to the midpoint *t*_*mid*_ over the study period is represented by t^*^ = *t* − *t*_*mid*_. The calculated spatial-temporal variability in disease risk in this model is decomposed in the following way:

The temporal term, (*b*_0_t^*^ + *v*_*t*_), indicates the overall time trend for all counties, defined as a linear trend, *b*_0_t^*^, and additional Gaussian noise, *v*_*t*_. Specifically, the term *b*_*1i*_t^*^ represents the departure from *b*_0_ for each county, which allows for each county to have its own trend, while *b*_0_ measures the overall temporal change in disease risk. For example, a positive estimate for *b*_*1i*_ suggests that the local variation intensity is higher than the overall variation trend; conversely, a negative estimate for *b*_*1i*_ reveals that the local variation intensity is lower than the overall variation trend.

The term *x*_*vit*_ repressents the temperature variation variable for area *i* and month *t*, and *β*_*v*_ is the corresponding regression coefficient. The term *x*_*nit*_ represents the *n*-th of the other potential confounds; the regression coefficient of these explanatory variables is *β*_*n*_.

The Gaussian random noise variable is represented by *ε*_*1i*_, involving all the factors that are not considered in the model but affect the explanatory variable, which is assumed to follow a normal distribution. That is, the Gaussian noise *ε*_*it*_ is modeled as *ε*_*it*_ ~N (0, *σ*_*ε*_^2^), and the temporal noise as *v*_*t*_ ~ N (0, *σ*_*v*_^2^). As suggested by Gelman, in this model, the prior distribution of the SDs (e.g., *σ*_*v*_, *σ*_*ε*_) of all the random variables is determined as a strictly positive half Gaussian distribution N_+∞_(0, 0.1).

In this study, the Besag, York, and Mollie (BYM) spatial model was introduced to determine the prior distribution of the parameters *s*_*i*_ and *b*_*1i*_^35,36^. It is a convolution of a spatially unstructured random effect and a spatially structured random effect, and can be expressed as follow:

3 $$\log ({\mu }_{i})={\rm{\alpha }}+{u}_{i}+{v}_{i}+f({c}_{i})$$

where the *u*_*i*_ represents unstructured random effects, and the *v*_*i*_ represents spatially structured heterogeneity, *f*(*c*_*i*_) is the non-linear effect of acovariate *c*_*i*_.

And the conditional autoregressive (CAR) prior is used to enhance the random effect of spatial structure in BYM with a spatial adjacency matrix W. The CAR prior on the spatial random effect indicates that adjacent counties tend to have similar disease risks.

All parameters were implemented in WinBUGS^37^, a statistical software package that was designed specifically for Bayesian calculations. Posterior distributions of all parameters in the model were obtained through Markov chain Monte Carlo (MCMC) simulations.

## Supplementary information

Supplementary Information.

## Data Availability

The datasets used and/or analyzed during the current study are available from the corresponding author on reasonable request.
