# Supplementary material for: Effects of temperature fluctuations on spatial-temporal transmission of hand, foot, and mouth disease
Source: Sci Rep. 2020 Feb 13;10:2541. doi: 10.1038/s41598-020-59265-z (PMC7018740; doi:10.1038/s41598-020-59265-z)
Supplement: Supplementary file 1 — Supplementary Information. [file 41598_2020_59265_MOESM1_ESM.pdf]

Supplementary Information

Title:

Effects of temperature fluctuations on spatial-temporal transmission of hand, foot, and mouth disease

Author names and affiliations:

Chengdong Xu[1#], Xiangxue Zhang[1,2#], Li Wang[3,4], YukeZhou[5], Gexin Xiao*[6,7], Jiaqiang Liao*[8]

[1]State Key Laboratory of Resources and Environmental Information System, Institute of Geographic Sciences and Natural Resources Research, Chinese Academy of Sciences, Beijing100101, China

[2] State Key Laboratory of Earth Surface Processes and Resource Ecology, Beijing Normal University, Beijing100875, China

[3]College of Environment and Planning, Henan University, KaiFeng475001, China.

[4]Key Laboratory of Geospatial Technology for the Middle and Lower Yellow River Regions (Henan University), Ministry of Education, Kai Feng 475001, China.

[5]Key Laboratory of Ecosystem Network Observation and Modeling, Institute of Geographic and Nature Resources Research, Chinese Academy of Sciences, Beijing 100101, China

[6]China National Center for Food Safety Risk Assessment, Beijing100022, China

[7]National Health Commission of the People's Republic of China, Beijing102206, China

[8]School of Public Health, Tongji Medical College, Huazhong University of Science and Technology, Wuhan430074, China

[*]Correspondence and requests for materials should be addressed to G.X. (email: biocomputer@126.com);J.L. (email:ljq19861023@163.com)

[#]Contributed equally.

Table S1 Quantified posterior means and RR of coefficients in BSTHM of Beijing.

Table S2 Quantified posterior means and RR of coefficients in BSTHM of Tianjin.

Table S3 Quantified posterior means and RR of coefficients in BSTHM of Hebei province.

Table S1 Quantified posterior means and RR of coefficients in BSTHM of Beijing.

Variables	Posterior mean (95% CI) (%)	RR (95% CI)
SD of maximum temperature(°C)	33.06** (5.74,60.77)	1.39 (1.06,1.84)
SD of minimum temperature(°C)	-1.59 (-23.22,21.42)	0.98 (0.79,1.24)
SD of average temperature(°C)	-19.30 (-59.66,20.35)	0.82 (0.55,1.23)
Average temperature (°C)	9.31** (6.87,12.01)	1.10 (1.07,1.13)
Relative humidity (%)	3.83** (2.26,5.79)	1.04 (1.02,1.06)
Wind speed (m/s)	40.70** (10.52,70.18)	1.50 (1.11,2.02)
Sun hours (h)	0.54** (0.09,1.04)	1.005 (1.00,1.01)
Precipitation (mm)	-0.07 (-0.28,0.14)	0.999 (0.997,1.00)

Note: 95% CI indicates confidence interval with a confidence level of 0.95,

**statistical significance level: 0.01.

Table S2 Quantified posterior means and RR of coefficients in BSTHM of Tianjin.

Variables	Posterior mean (95% CI) (%)	RR (95% CI)
SD of maximum temperature(°C)	-10.09 (-69.92,53.49)	0.90 (0.50,1.71)
SD of minimum temperature(°C)	-21.09 (-75.79,31.60)	0.81 (0.47,1.37)
SD of average temperature(°C)	47.59 (-52.18,142.00)	1.61 (0.59,4.14)
Average temperature (°C)	11.21** (8.53,13.99)	1.12 (1.09,1.15)
Relative humidity (%)	0.76 (-1.74,3.03)	1.01 (0.92,1.03)
Wind speed (m/s)	27.97 (-8.95,65.05)	1.32 (0.91,1.92)
Sun hours (h)	0.07 (-0.49,0.59)	1.00 (0.99,1.01)
Precipitation (mm)	0.62** (0.29,0.95)	1.006 (1.00,1.01)

Note: 95% CI indicates confidence interval with a confidence level of 0.95,

**statistical significance level: 0.01.

Table S3 Quantified posterior means and RR of coefficients in BSTHM of Hebei province.

Variables	Posterior mean (95% CI) (%)	RR (95% CI)
SD of maximum temperature(°C)	-6.45 (-19.78,5.94)	0.94 (0.82,1.06)
SD of minimum temperature(°C)	-8.92 (-22.80,3.67)	0.91 (0.80,1.04)
SD of average temperature(°C)	1.85 (-16.90,22.53)	1.02 (0.84,1.25)
Average temperature (°C)	19.95** (17.72,22.33)	1.22 (1.19,1.25)
Relative humidity (%)	4.25** (3.71,5.04)	1.04 (1.03,1.05)
Wind speed (m/s)	-26.27** (-38.23,-14.29)	0.77 (0.68,0.87)
Sun hours (h)	0.02 (-0.13,0.16)	1.00 (0.999,1.002)
Precipitation (mm)	-0.19** (-0.27,-0.11)	0.998 (0.997,1.00)

Note: 95% CI indicates confidence interval with a confidence level of 0.95,

**statistical significance level: 0.01.
